# Use of health services among international migrant children – a systematic review

**DOI:** 10.1186/s12992-018-0370-9

**Published:** 2018-05-16

**Authors:** Niina Markkula, Baltica Cabieses, Venla Lehti, Eleonora Uphoff, Sofia Astorga, Francisca Stutzin

**Affiliations:** 1 Social Studies in Health Research Programme, Instituto de Ciencias e Innovación en Medicina (ICIM), Facultad de Medicina, Clínica Alemana Universidad del Desarrollo, Av. Las Condes 12461, Las Condes, Santiago Chile; 2 Department of Health Sciences, University of York, York, England; 3 Department of Psychiatry, Helsinki University Hospital and University of Helsinki, Helsinki, Finland; 4 Centre for Interdisciplinary and Intercultural Inquiry, Health Humanities, University College London, London, UK

**Keywords:** Transients and migrants, Immigrants, Children, Health service use, Access

## Abstract

**Background:**

Migrant children have specific health needs, and may face difficulties in accessing health care, but not enough is known about their health service use. This study aims to describe patterns of use of health services of international migrant children and differences to respective native populations.

**Methods:**

Electronic databases PubMed and Web of Science, references of identified publications, and websites of relevant international agencies were searched. We included observational studies published between 2006 and 2016 that reported use of formal health services by migrant children (0–18 years), including first and second generation migrants. Data on study characteristics, study theme, main outcome and study quality were extracted.

**Results:**

One hundred seven full texts were included in the review. Of the studies that reported comparable outcomes, half (50%) indicated less use of healthcare by migrants compared with non-migrants; 25% reported no difference, 18% reported greater use, and 7% did not report this outcome. There was variation by theme, so that the proportion of conclusions “less use” was most common in the categories “general access to care”, “primary care” and “oral health”, whereas in the use of emergency rooms or hospitalisations, the most common conclusion was “greater use”.

**Conclusions:**

Migrant children appear to use different types of healthcare services less than native populations, with the exception of emergency and hospital services.

**Systematic review registration:**

PROSPERO systematic review registration number: CRD42016039876.

**Electronic supplementary material:**

The online version of this article (10.1186/s12992-018-0370-9) contains supplementary material, which is available to authorized users.

## Background

International migration is a global concern and a well-known social determinant of health [1, 2]. Migration as a phenomenon has health impacts both at the individual and population level [3–5]. However, the recognition of migration as a social determinant of health is not straightforward, as this implies taking a moral stance regarding values of ethics and fairness in policy making [6]. There were 258 million international migrants in the world in 2017, 49% more than in the year 2000 [7]. Migrants represent 3.4% of the world’s population and 14% of the population living in high-income countries. One in ten migrants is a refugee [7]. In October 2016, the New York Declaration of the United Nations General Assembly urged all countries to protect the human rights of all refugees and migrants, placing special emphasis on vulnerable groups such as migrant children [8]. The health sector has been criticised for its slow response to the commitments of the assembly [9].

It has been estimated that 37 million international migrants are children, including 11 million refugees and asylum seekers [10]. In the past 10 years, the number of child refugees has more than doubled [10]. All children, including international migrants, have a human right of access to health care facilities that allow them to enjoy the highest attainable standard of health [11]. Refugee and asylum seeking children who may have had a dangerous journey and face adverse living conditions are particularly vulnerable [12]. International migrant children need specific attention and there is an opportunity for healthcare systems, amongst other social structures, to address the protection and recovery of their wellbeing and health throughout their life course [13].

International migrant children and youth face different health challenges compared with local populations due to the psychosocial stress of the migration process, adverse social conditions and increased exposure to health risks [1, 14, 15]. The available literature shows differences in health status of migrant children from the moment of birth: children born to mothers with immigrant background have a higher incidence of stillbirth, neonatal death, premature delivery and low birth weight [16, 17]. Among young children, higher rates of dental cavities, some infectious diseases and obesity have been found [17, 18]. Also higher rates of some mental disorders have been reported [19–21].

Systematic reviews on the use of health services among adult migrants have found varying patterns of health care use, so that the use of preventive services is lower than among the general population, but the use of primary care and rate of hospitalisations is higher [16, 22–24]. However, these findings cannot be generalized to children. In the case of children, the decision-making involves also their parents or other caretakers, and possibly other actors. Few available studies suggest that language barriers [25] and parents’ expectations [15] are particularly relevant determinants of access to healthcare. Large differences have been found in health service use depending on the type of service and origin of the children, making it difficult to extract information to support health policy decision-making [17, 26].

With the increase in international migration [7], it seems relevant to collect and systematize pertinent information on health service use of migrant children. This systematic review aims to describe use of health services of international migrant children and possible differences to respective local populations in different healthcare settings.

## Methods

### Type of study

We conducted a systematic review in accordance with the guidelines set by the Preferred Reporting Items for Systematic Reviews and Meta-Analyses (PRISMA) statement. The study protocol was registered at PROSPERO in May 2016 (http://www.crd.york.ac.uk/PROSPERO/display_record.php?ID=CRD42016039876, registration No. CRD42016039876).

### Search strategy

**Fig. 1:**
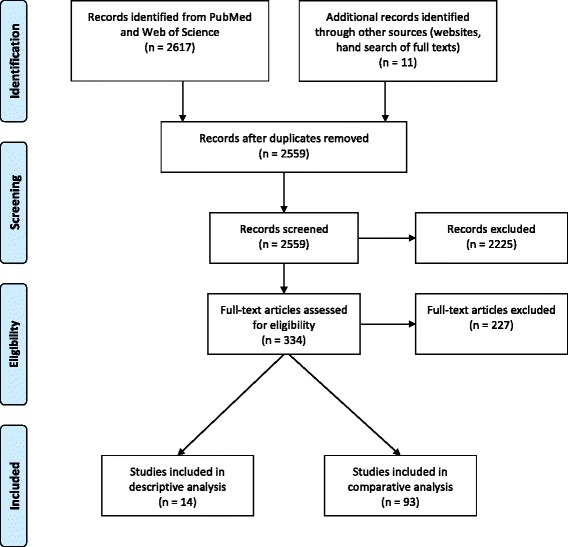
PRISMA flow diagram of the study screening and selection process

We searched PubMed and Web of Science electronic databases using the search terms specified in Table 1, for publications published in January 2006 to May 2016. This timeframe was chosen because changes in patterns of migration may have influenced access in recent decades and the aim was to analyse the current situation, which was considered more useful to orient health policies. A filter for observational studies was used as detailed in Table 1. The database search was carried out in May 2016 (Fig. 1). The search in PubMed yielded 1912 hits and Web of Science 705 hits. Additionally, we searched the website of the International Office for Migration (IOM). This search yielded one additional publication.

**Table 1:** Search strategy

*For general health services use:* (“Health Services”[Mesh] OR “health care” OR “Health service use” OR “Health service utilization” OR “Health care use” OR “Healthcare use” *For specific health services use:* OR “Specialised health services” OR “Specialist” OR “Hospitalisation” OR “Emergency health services” OR “Mental health services” OR “Preventive health services” OR “health check-ups” OR “primary service” OR “dental care” OR “dental”) AND	
(“Transients and Migrants”[Mesh] OR “Emigrants and immigrants”[Mesh] OR “Refugee” OR “Migration background” OR “Immigrant background” OR “Migrant” OR “Migrants” OR “Immigrant” OR “Immigrants” OR “Ethnic minority”) AND	
(“Child”[Mesh] OR “Children” OR “Adolescent” OR “Adolescents” OR “Youth” OR “minor”) AND	
(“cohort studies”[Mesh] OR “case-control studies”[mesh] OR “comparative study”[pt] OR “risk factors”[mesh] OR “cohort”[tw] OR “compared”[tw] OR “groups”[tw] OR “case control”[tw] OR “multivariate”[tw])

### Inclusion and exclusion criteria

Study inclusion criteria were 1) Study population included international migrant children and adolescents aged less than 18 years old. First and second-generation migrants were included. If the study reported a wider age range, studies were included only if they reported results separated for children; 2) The study methodology was quantitative and observational, including cross-sectional, case-control and cohort studies; 3) The study reported on health service use. *Migrant* was defined as someone who has (or in the case of second-generation migrants, whose parents have) crossed a national border to reside in another country for a year or longer. Publications referring only to ethnic minorities without clearly stating the migration status of the participants were not included. All formal health services were included, whether primary, secondary, preventive or curative, public or private. All types of contact with health services were included, and “health service use” was conceptualized to encompass concepts such as effective use and utilization, and also having a usual source of care [27]. Use of prescription medication was considered to indicate use of health services. Studies analysing national migration, only analysing health insurance status and economic evaluations were excluded. Studies in English, Spanish, Portuguese, French, Dutch, Swedish and Finnish were included.

### Selection and retrieval process

Based upon selection criteria, two researchers (NM, BC) independently evaluated each title and abstract. Every abstract was marked as selected/ not selected/ unclear in an Excel sheet. In case of disagreement on inclusion and exclusion, as well as when a paper fell into the “unclear” category, a third person (VL) took a decision. After review, 324 titles were chosen for full text review by the same researchers. Full texts were reviewed to ensure the publication met the inclusion criteria. Altogether 97 full texts were selected. After this, the references of the selected full texts were hand searched, and an additional 10 publications were included, as they met the inclusion criteria. All chosen full texts were located through university libraries or by contacting the authors directly.

### Data extraction

The data was extracted in an Excel sheet (available upon request). Four trained researchers (NM, BC, SA, FS) participated in data extraction. After data from 10 papers was extracted, researchers met twice and discussed their strategies and concerns. This allowed controlling for differences in data extraction criteria and process between researchers (i.e. harmonization of this stage across individuals).

### Quality assessment

Quality of the studies was assessed using nine quality criteria derived from the Strengthening the Reporting of Observational Studies in Epidemiology (STROBE) and the Mixed Methods Appraisal Tool Version 2011 (MMAT-Version 2011) to assess each publication as part of the data extraction: study question is well justified; study has clear objectives or hypothesis; study design is clear; participants are well described; the sample is representative of population of interest; sample size is adequate; the main outcome is clearly described; analyses are well described, and results are adjusted by confounders. A score from 0 to 9 was assigned to each study based on the nine quality criteria, and studies that fulfilled 0 to 3 criteria were labelled ‘poor quality’, 4 to 6 was considered ‘average quality’ and 7 to 9 criteria met indicated ‘good quality’.

### Data analysis

In total, 107 full texts were included in the analyses. Fourteen publications that did not compare health care use of migrants with native populations were analysed separately from the 93 publications that did include a comparison to native populations.

Since there was significant heterogeneity in the themes, populations and results, a narrative synthesis instead of a meta-analysis was conducted. The main result was categorized into “lower utilization/access”, “higher utilization/access” and “no significant difference” in comparison to native populations. Data analysis also included the description of recipient continents and countries, study design, type of migrant children, origin of migrants, sample size, main outcome, and control variables reported in all selected papers. It also includes a description of risk of bias assessment. Additionally, sub-group analyses were conducted by study topic for the following main themes: vaccines; mental health; hospital/emergency room (ER) use; oral health; general access/use; primary care; and other.

## Results

### Description of comparative studies

**Fig. 2:**
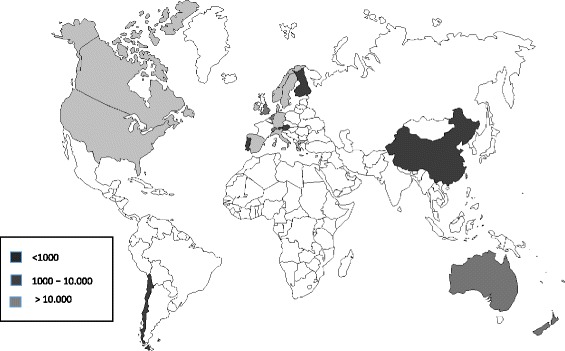
Recipient countries of immigrants by sample size. Darkest grey indicates a sample size < 1000 persons, medium grey 1000–10.000 persons and lightest grey > 10.000 persons

The 93 comparative studies, including 10.030.311 children, originated mostly from Europe (57%) and North America (36%) (Table 2, Fig. 2, Additional file 1: Table S1). The themes covered were general access or having a usual source of care (30%), vaccines (20%), mental health (18%), hospital or emergency room use (16%), oral health (14%) and primary care use (13%). Majority of the studies included large samples, with 40% having a sample size of over 10.000. Some 35% were nationally representative, and 47% utilized register data. Majority (77%) of the studies adjusted for confounding variables, most commonly sex (48%), age (47%) and indicators of socioeconomic status (16–23%).

**Table 2:** Description of the studies in the comparative analysis (*n* = 93)

	Number (%)
Continent of receiving country	
Europe	54 (58%)
North America	34 (37%)
Asia	3 (3%)
Australia	2 (2%)
Theme (combinations possible)	
General access/having a usual source of care	27 (29%)
Vaccines	19 (22%)
Mental health	16 (17%)
Hospital or ER use	15 (16%)
Oral health	14 (15%)
Primary care use	12 (13%)
Other	2 (2%)
Study population characteristics	
Sample size	
Sample size < 200	8 (9%)
Sample size 200–10.000	46 (50%)
Sample size > 10.000	39 (42%)
Type of migrants	
Not specified or mixed	85 (91%)
Refugee only	8 (9%)
Labour only	0
Generation of migrants	
Not specified or mixed	13 (14%)
Only first-generation migrants	16 (17%)
Only second-generation migrants	64 (69%)
Age range	
< 7 years only	14 (15%)
12–18 years only	11 (12%)
Other or all children 0–18 years	68 (73%)
Origin of migrants	
All or several countries	37 (40%)
Hispanic	6 (6%)
Asian	2 (2%)
African	1 (1%)
Non-Western/Less developed countries	3 (3%)
Western countries	1 (1%)
Turkey	1 (1%)
Chile	1 (1%)
North Korea	1 (1%)
Not specified	41 (44%)
Methodological characteristics	
Study design	
Cross-sectional	76 (82%)
Longitudinal (prospective, retrospective)	17 (18%)
Study representativeness	
Regional	60 (65%)
National	33 (35%)
Data source (combinations possible)	
Register or other routine data	44 (47%)
National survey	29 (31%)
Questionnaire to a targeted study population	26 (28%)
Other	7 (8%)
Type of source (combinations possible)	
Register or other routine data	39 (42%)
Parent-report	36 (39%)
Self-report	13 (14%)
Other (e.g. blood sample)	7 (8%)
Confounding variables adjusted for (combinations possible)	
Sex	45 (48%)
Age	44 (47%)
Education of parents	22 (24%)
Income of parents	15 (16%)
Other SES of parents	15 (16%)
Need indicators (health status)	15 (16%)
Insurance	14 (15%)
Ethnicity	10 (11%)
Language spoken at home	6 (6%)
Parental attitudes or beliefs	5 (5%)
None	24 (26%)

**Fig. 3:**
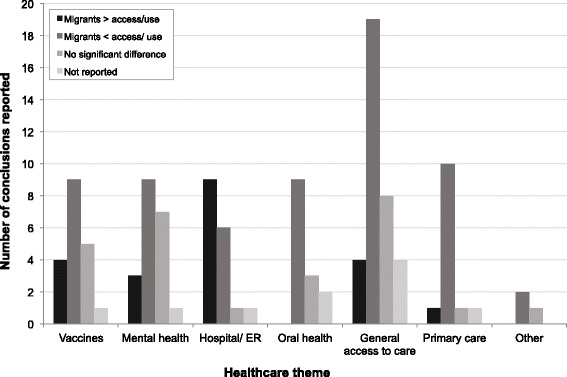
Main conclusions of studies by healthcare theme

The 93 studies reported in total 123 outcomes that could be categorised into “higher utilization/access”, “lower utilization/access”, “no significant difference” and “not reported”. Half (50.4%) of these outcomes indicated lower utilization of healthcare by migrants compared with non-migrants; 25.2% reported no difference, 17.9% reported higher use, and 6.5% did not report this outcome (Additional file 2: Table S2, Fig. 3). Analysing this by theme, the proportion of conclusions “lower utilization” was most common in the categories “general access to care”, “primary care”, “oral health”, “vaccines” and “mental health”, whereas in the use of hospital or ER services the most common conclusion was “higher utilization”.

### Studies without a comparison group

The 14 studies without comparison group (Additional file 2: Tables S2-S3) did not significantly differ in methodological characteristics, with the exception of smaller sample size, more frequent regional or local representativeness, and more frequent use of questionnaire as opposed to survey or register data. Two Australian studies without comparison group studied vaccines: the immunization coverage in immigrant children from East Africa [28] was found unknown or incomplete in 97%. Another study reported on a school-based vaccination programme targeted for migrant youth, who had a low initial coverage (30% for MMR and 18% for hepatitis B) [29]. Three studies reported on mental health, discovering high rates of unmet mental health needs in Chile [30] and US [31], but also high rates of mental health counselling received by unaccompanied Sudanese minors in the US [32]. Two studies analysed use of dental services [33, 34], finding suboptimal rates, and six studies reported on rates and diagnoses of primary care or hospital use [35–40].

The only study comparing migrants with the national population of the country of origin [41], analysed as part of the studies without comparison group since it did not include the national population, found lower rates of asthma but higher rates of infectious diseases among Japanese children living in Thailand than those living in Japan.

### Quality of studies

Twelve studies (13%) were considered of average quality and 82 (87%) studies of good quality. No study was of poor quality. Of the quality criteria utilized, most frequently adjustment for confounders was missing (24 studies), followed by unclear study design (16 studies), inadequate sample size and unclear analysis (both 14 studies). Four out of twelve average-quality studies were national publications from Spain and Portugal; altogether nine were European, two North American and one was a South American publication.

### Sensitivity analysis with high quality studies

Twelve studies of average quality were excluded to perform a sensitivity analysis with high quality studies only (available upon request). The results confirm findings of the complete analysis, with 50.0% of reported conclusions pointing towards less access or use of healthcare by migrants compared to non-migrants, and 20.2% concluding that access or use of healthcare was greater for migrants.

## Discussion

### Main findings

This systematic review identified 107 studies reporting on healthcare use of migrant children, published from 2006 to 2016. To the best of our knowledge, it is the first attempt to systematize scientific knowledge on patterns of healthcare use among migrant children, a growing group with specific health and healthcare needs. The identified studies originated mostly from Europe and North America, with only 7% of studies coming from other regions. While these two regions host just over half of global international migrants [42], among children the distribution is different: three out of five child migrants live in Asia and Africa [10]. There is a clear lack of studies from these regions with large migrant populations.

Methodologically the studies appear strong, with 87% categorized as meeting good quality criteria. Most studies had large sample sizes, one third of them were nationally representative, and a large majority adjusted for important confounding variables. However, majority of studies did not adjust for socioeconomic status, which could be problematic. Even fewer studies adjusted for indicators of need, such as health status, which should be considered when interpreting the results: migrant and native children may have differing healthcare needs, leading to different utilization patterns.

The studies focused on a few main themes, such as vaccinations, and oral and mental health, exploring both primary and specialized care use. Studies from the US frequently assessed existence of a usual source of care. Besides vaccinations and dental check-ups, no other aspects of preventive care or health promotion were studied. This is particularly noteworthy, since some migrant children in Europe have higher rates of risk factors such as obesity and physical inactivity than native children, which highlights the importance of health promotion in these groups [18]. Among migrant women, attendance to prenatal check-ups has been studied extensively and found generally to be lower than in native populations [16], and therefore preventive care use merits attention among migrant children as well.

Only 9% (8 studies) of the identified studies focused on refugees, and three additional studies included refugees. One of these studies was carried out in Asia [43], one in Australia [44], three in North America [37, 45, 46] and six in Europe [47–52]. In total, four studies found higher use of health services among refugees, four found lower service use and three found either no difference or they did not compare. As refugee children are a very specific and growing group [10], and findings regarding health service use among other type of migrants may not be applicable to them, the low number of studies on refugees brings to question what is really known about health service use of this particular group of children.

When analysing the main results by healthcare theme, the most striking difference is the relatively frequent finding of “higher use or access” in the category hospital and emergency room services. Majority of the studies that found higher use in this category originated from Europe [53–59], while two studies originated from North America [45, 60]. Two of the studies included refugees only, and three examined risk of hospitalization among children with type 1 diabetes [54, 55, 57]. A Swiss study found that migrants were overrepresented at the paediatric intensive care unit [59]. Therefore, several of these studies appear to indicate delayed care or problems in accessing routine treatment, rather than overuse.

### Possible reasons to reduced utilization and access

Cultural norms, explanatory models of disease, lack of safety networks, language barriers and economic and social adversity all interplay in migrants’ decision to seek and use health services [61]. The reasons for differing use of health services among migrant children could be categorized into family-related (such as fear, stigma, lack of trust, financial difficulties, problems in navigating new healthcare systems, lack of awareness of rights); those related to health professionals (communication problems, misconceptions, cultural barriers), and structural problems related to healthcare systems (lack of entitlement to care or restrictions to use, problems in physical access) [62].

These factors affect migrant families of different characteristics to varying degrees: Among migrants with undocumented immigration status, lack of awareness of their rights and functioning of health care systems, fear, and economic reasons may be the most important [63]. For refugees and asylum seekers, barriers to care are often related to legal entitlement, but organizational barriers and lack of provider expertise also influence their access to care [64]. Other barriers identified as important for labour-migrants in particular include lack of health insurance, lack of awareness about occupational health and safety regulations, and documentation status [62]. Several other issues have also been identified as influencing access among migrants: physically moving from one place to another, thereby discontinuing any on-going treatment or vaccine series; lack of coordination among the health authorities inside and between countries; and lack of resources in the hosting countries [24].

A potential factor influencing access of all migrants and ethnic minorities are health care professionals’ skills and attitudes. A systematic review identified three main components of this barrier: biases, stereotypes and prejudices; language and communication barriers, and cultural misunderstandings [65]. Another systematic review concludes that this type of implicit bias is likely to influence clinical decisions [66].

Finally, different use of health services may also result from different needs. While this is likely not true for lower vaccination rates or lower use of dental care, it may explain to some extent lower use of other services, where the need appears to be less than in native populations. The ‘healthy migrant effect’ has been observed in rates of asthma, some mental problems and risky health behaviours, which all appear to be lower than among native populations [13, 67].

### Comparison to other studies

Another systematic review on adult migrants’ health service use, limited to use of somatic services and the European region [23], found more varying results than our review. Use of preventive services such as mammography screening was lower, whereas use of general practitioners’ services and rate of hospitalizations was higher. A recent systematic review focused on the use of emergency department services in Europe found higher and sometimes inappropriate use among migrants [68]. One explanation proposed by the authors is difficulties in accessing more appropriate sources of health care.

Also in line with our findings, a systematic review on vaccination coverage of rural-urban migrant children found a lower rate among migrants than the general population [69]. Similarly, a systematic review on vaccine coverage of migrant and refugee adults in Europe found lower coverage than among native populations [24].

### Strengths and limitations

To our knowledge, this is the first effort to systematize published research on the use of health services of international migrant children. We used a broad search strategy and found a large number of studies, reporting on more than 10 million children.

However, the study has certain limitations that should be considered. The identified studies were heterogeneous, which makes interpretation of the results more challenging, and also prevented us from carrying out a meta-analysis. Unfortunately, information on countries of origin of the migrants was not available for many studies, and it was not possible to analyse studies by subgroups based on country of origin. Also analyses by type of migrants were not possible because this information was frequently lacking, and number of studies in each group was small. Our review was limited to literature published between 2006 and 2016 and to two databases, and therefore possibly relevant literature could have been missed. Findings published in reports or in languages other than the ones included could also be relevant, and were not included in this study. Most studies originated from Europe and North America, and therefore we cannot draw firm conclusions on migrant children settled in other regions of the world. Some studies were not originally designed to address research questions about migrants or not focused on children, which could also be considered a limitation.

Additionally, it should be noted that the comparison between migrants and native populations does not take into account suboptimal access of native children [70–72]. Nevertheless, in a review the comparison to native populations is the clearest method to point out inequalities in service use between these two groups, even though both may have problems in accessing health care. Further, this study only addressed health service use in the post-migratory situation, leaving out many significant variables that affect why, how and when they migrated, as well as what patterns of access to healthcare existed in their countries of origin. By systematizing several studies, this review overlooks the unique characteristics of individual studies and contexts. To include these complexities would be very hard to disentangle in a single systematic review, and therefore this study can be considered a baseline for further studies, and as such aims to analyse the general tendencies of patterns of use of healthcare among migrant children. International migration should be studied at the local, national, regional and global scales, as this phenomenon responds to complex and dynamic processes of globalization, international labour stratification, poverty and conflict. This paper is unable to mirror all these factors, but they could be studied using different methodologies than a systematic review.

### Implications

Migrant children have reduced use of many types of health services. According to our findings, only the use of emergency and hospital services was found to be higher than native populations, which appears to indicate problems in accessing care at earlier stages or more appropriate places.

Targeted policies could help overcome these barriers in access to healthcare. For example, improving health literacy seems to have the potential to empower patients and reduce health inequalities, and there are effective interventions to improve health literacy among migrants [73]. Regarding health providers, some of the identified barriers could be ameliorated with system changes, such as utilization of interpreters, whereas others would require specific interventions such as cultural competence education for health professionals [74]. Recently, interventions to improve immigrant health were reviewed, and many specific policies for adult migrants were identified. However, only 11% of the results were policies directed to children [75]. There appears to be a need to develop and document policies to improve access to care for child migrants.

Future research should extend beyond Europe and North America to the regions with most child migrants: Asia, Africa and South America. Moreover, while the identified studies were large and used reliable methods, the majority did not control for measures of socioeconomic status, and few were able to control for indicators of need, such as health status. It is important to develop study methodologies that can better control for confounding factors, and more precisely measure the impact of migration on service use. Since migrant groups are different in terms of their health care needs, reasons for migration, region of origin and time since arrival should be documented more carefully both in registers and in studies, to identify and analyse groups that have a particularly high risk of underuse of services. Finally, the reasons and mechanisms for foregone and delayed care should be also evaluated in epidemiological studies.

## Conclusion

Children of international migrants use most types of healthcare services less than local children: they are less likely to have a usual health service provider, to use preventive services, primary and dental care, and some specialised health services. Considering the risk that international migration presents to health, these findings warrant action both to ensure equitable access to health services, and to safeguard the right to health for all children.

## Additional files

Additional file 1: **Table S1.** Results of the studies with comparison group (*n* = 93). (XLSX 50 kb)

Additional file 2: **Table S2.** Healthcare use by migrant children: number of main conclusions reported by theme. **Table S3.** Description of the studies without comparison group (*n* = 14). **Table S4.** Results of the studies without comparison group (*n* = 14). (DOCX 75 kb)
