# Supplementary material for: Use of health services among international migrant children – a systematic review
Source: Global Health. 2018 May 16;14:52. doi: 10.1186/s12992-018-0370-9 (PMC5956827; doi:10.1186/s12992-018-0370-9)
Supplement: Supplementary file 2 — Table S2. Healthcare use by migrant children: number of main conclusions reported by theme. Table S3. Description of the studies without comparison group (n = 14). Table S4. Results of the studies without comparison group (n = 14). (DOCX 75 kb) [file 12992_2018_370_MOESM2_ESM.docx]

**Additional file 2: Table S2. Healthcare use by migrant children: number of main conclusions reported by theme**

| Healthcare theme | Greater access or use of healthcare by migrants than non-migrants | Less access or use of healthcare by migrants than non-migrants | No significant difference in healthcare use for migrants vs. non-migrants | Result on healthcare use for migrants vs. non-migrants not reported | **TOTAL number of conclusions reported (% of column total)*** |
| --- | --- | --- | --- | --- | --- |
| 1 Vaccines | 4 | 9 | 6 | 0 | **19 (15.4%)** |
| 2 Mental health | 3 | 9 | 8 | 0 | **20 (16.3%)** |
| 3 Hospital/ ER | 9 | 6 | 1 | 1 | **17 (13.8%)** |
| 4 Oral health | 1 | 9 | 4 | 2 | **16 (13.0%)** |
| 5 General access | 4 | 18 | 9 | 4 | **35 (28.4%)** |
| 6 Primary care | 1 | 9 | 2 | 1 | **13 (10.7%)** |
| 7 Other | 0 | 2 | 1 | 0 | **3 (2.4%)** |
| **TOTAL number of conclusions reported (% of row total)*** | **22 (17.9%)** | **62 (50.4%)** | **31 (25.2%)** | **8 (6.5%)** | **123 (100%)** |

* One study may report on more than one healthcare theme, and one study may draw different conclusions based on population, healthcare theme and outcome studied.

**Additional file 2: Table S3. Description of the studies without comparison group (n=14)**

|  | Number (%) |
| --- | --- |
| **Continent of receiving country** |  |
| Europe | 3 (21.4) |
| North America | 6 (42.9) |
| Asia | 1 (7.1) |
| Australia | 3 (21.4) |
| South America | 1 (7.1) |
| **Theme** (combinations possible) |  |
| General access / having a usual source of care | 4 (28.6) |
| Vaccines | 2 (14.3) |
| Mental health | 3 (21.4) |
| Hospital or ER use | 3 (21.4) |
| Oral health | 2 (14.3) |
| Primary care use | 1 (7.1) |
| Other | - |
| **Sample size** |  |
| Sample size <200 | 6 (42.9) |
| Sample size 200-10.000 | 6 (42.9) |
| Sample size >10.000 | 2 (14.3) |
| **Type of migrants** |  |
| Not specified or mixed | 11 (78.6) |
| Refugee only | 3 (21.4) |
| Labour only |  |
| **Generation of migrants** |  |
| Not specified or mixed | 9 (64.3) |
| Only first-generation migrants | 4 (28.6) |
| Only second-generation migrants | 1 (7.1) |
| **Age range** |  |
| <7 years only | 1 (7.1) |
| 12-18 years only | 0 |
| Other or all children 0-18 years | 13 (92.9) |
| **Study design** |  |
| Cross-sectional | 11 (78.6) |
| Longitudinal (prospective, retrospective) | 2 (14.3) |
| Other (before-after comparison) | 1 (7.1) |
| **Study representativeness** |  |
| Regional | 11 (78.6) |
| National | 3 (21.4) |
| **Type of sample** |  |
| Purposive | 6 (42.9) |
| Random | 3 (21.4) |
| Convenience | 3 (21.4) |
| Other | 2 (14.3) |
| **Data source** (combinations possible) |  |
| Register or other routine data | 4 (28.6) |
| National survey | 1 (7.1) |
| Questionnaire | 9 (64.3) |
| Other | 1 (7.1) |
| **Type of source** (combinations possible) |  |
| Register or other routine data | 4 (28.6) |
| Parent-report | 7 (50.0) |
| Self-report | 3 (21.4) |
| Other (e.g. blood sample) | 1 (7.1) |
|  |  |

**Additional file 2: Table S4. Results of the studies without comparison group (n=14)**

| **Publication information** | **Study objective** | **Continent** | **Main study outcome** | **Main result** |
| --- | --- | --- | --- | --- |
| **Vaccines** |  |  |  |  |
| Milne B, Raman S, Thomas P, et al.: Immunisation of refugee and migrant young people: can schools do the job? Australian and New Zealand journal of public health 30:526-8, 2006 | To assess immunisation needs, primary health care (PHC) use and trial a school-based immunisation service for refugee and migrant young people attending an Intensive English Centre (IEC) high school in Western Sydney. | Australia | Immunisation rate for MMR and hepatitis B, visiting a doctor in the past year | Forty-nine students (30%) reported previous immunisation with MMR and 29 (18%) with hepatitis B. As part of the school immunisation program, 142 (74%) received MMR vaccine, 151 (78%) received the first dose of hepatitis B vaccine, 144 (95%) received the second dose of hepatitis B, and 34 (23%) received the third hepatitis B dose elsewhere. |
| Paxton GA, Rice J, Davie G, et al.: East African immigrant children in Australia have poor immunisation coverage. J Paediatr Child Health 47:888-92, 2011 | To provide data on the immunisation status of recently arrived East African children and adolescents in Australia. | Australia | Immunisation rate for tetanus, BCG, measles, diphtheria, rubella, hepatitis B, hip and oral polio vaccine | 97% had incomplete or unknown immunisation status based on parent report and vaccination records. Only 15% had serological immunity to all five of measles, rubella, tetanus, diphtheria and hepatitis B |
| **Mental health** |  |  |  |  |
| Rojas G, Fritsch R, Castro A, et al.: Mental disorders among immigrants in Chile. Revista Medica De Chile 139:1298-304, 2011 | To determine the prevalence of Common Mental Disorders (CMD) among immigrants who live in Independencia, a municipality in Santiago, Chile. | South America | Use of and barriers to access for mental health services | 32.4% (according to pupils) or 36.1% (according to parents) of school children have a psychiatric problem for which they consider they need help, but only 9.6% are currently receiving psychiatric treatment. Main reasons include worrying about cost of treatment, thinking the problem would resolve on its own and not knowing how the health care system works. |
| Toppelberg CO, Hollinshead MO, Collins BA, et al.: Cross-Sectional Study of Unmet Mental Health Need in 5- to 7-Year Old Latino Children in the United States: Do Teachers and Parents Make a Difference in Service Utilization? School mental health 5:59-69, 2013 | Examine the rates of mental health service utilization in young Latino children of immigrants in relation to maternal and teacher reports of child mental health need | North America | Mental health need type and mental health service received | High rates of unmet mental needs: 28.5 % of the immigrant children had a mental health need; 76.9 % of these received no services. |
| Geltman PL, Grant-Knight W, Ellis H, et al.: The "lost boys" of Sudan: use of health services and functional health outcomes of unaccompanied refugee minors resettled in the U.S. Journal of immigrant and minority health / Center for Minority Public Health 10:389-96, 2008 | To assess whether mental health counseling and other health services were associated with functional health outcomes of unaccompanied Sudanese refugee | America | Mental health counseling and other health services | Minors reported high rates of counseling (45%); however no differences were noted in counseling use by those with PTSD compared with other health outcomes. |
| **Hospital or ER use** |  |  |  |  |
| del Rosario CR, Diaz SN, de Carlos PG, et al.: Health care for African immigrants arriving in the Canary Islands a descriptive study. Emergencias 20:411-8, 2008 | The aim of this study was to describe the characteristics of health care provided to arriving African immigrants, including primary care, hospital emergency care, and hospital admissions | Europe | Characteristics of provided health care | The most frequent diagnoses amongst immigrants were related to infectious and parasitic diseases, among which malaria by Plasmodium falciparum represented a total of 228 stays. The boat crossings of thousands of kilometers affect the health of many immigrants to greater or lesser degrees. |
| Sakai R, Wongkhomthong SA, Marui E: Disease patterns of outpatient visits by Japanese expatriate children in Thailand. Acta paediatrica (Oslo, Norway : 1992) 98:573-8, 2009 | To clarify the health-related conditions of Japanese expatriate children in Thailand. | Asia | Difference in prevalence of diseases between Japanese children living in Thailand and in Japan | Children living in Japan contract asthma (22,6%) more frequently than infectious diseases (6%), whereas those living in Thailand show the opposite trend (1,5% for asthma, 12,1% for infectious diseases). |
| Sabbatani S, Baldi E, Manfredi R: Causes of hospitalization among extra-European Union children in a large hospital of Northern Italy, in a five-year observation period. The Brazilian journal of infectious diseases : an official publication of the Brazilian Society of Infectious Diseases 11:6-8, 2007 | Assess all hospitalizations carried out at our tertiary care reference Hospital (S. Orsola-Malpighi Hospital, Bologna, Italy), from the year 1999 (January 1) to 2004 (December 31), by extra-European Union (EU) children aged 14 years or less, in order to indirectly evaluate the prominent health problems of young immigrants and their children, by looking at admissions, discharge diagnoses, and their multiple correlates | Europe | Difference in the admission rates to hospital, before and after the 2001 law | Increased number of admissions was observed since the year 2002 (chi-square 268.107; p<.001). This increase was mainly attributable to Eastern European children (44.1%) |
| **Oral health** |  |  |  |  |
| Nahouraii H, Wasserman M, Bender DE, et al.: Social support and dental utilization among children of Latina immigrants. Journal of health care for the poor and underserved 19:428-41, 2008 | To determine whether four types of social support (information, influence, material aid, emotional aid) could mitigate barriers to pediatric dental care. | North America | Association between four types of social support and dental care use among children of Latina immigrants | The majority of children in this study had visited a dentist at some point in their lives (57.0%). Most mothers (58.0%) described the condition of her index child’s teeth as excellent, very good, or good, while 42.0% described it as so-so or bad. Compared with children whose mothers had received no social support, the odds of having had a dental care visit were 3.1 times greater (95% CI 5 2.21–6.60) for children whose mothers had received some social support. |
| Quandt SA, Clark HM, Rao P, et al.: Oral health of children and adults in Latino migrant and seasonal farmworker families. Journal of immigrant and minority health 9:229-35, 2007 | To describe use of dental services of children and parents in farmworker families | America | Use of dental services | Dental care had been received in the past year by 73% of children, 47% of mothers, and 37% of spouses. |
| **General access and primary care** |  |  |  |  |
| Watts DJ, Friedman JF, Vivier PM, et al.: Health care utilization of refugee children after resettlement. Journal of immigrant and minority health / Center for Minority Public Health 14:583-8, 2012 | To assess health care utilization of refugee children after resettlement. | America | Health status and health care utilization | After arrival, 21% had an emergency department visit, 40% had a primary care sick visit, and 71% had a primary care follow-up. |
| Thomas P, Milne B, Raman S, et al.: Refugee youth--immunisation status and GP attendance. Australian family physician 36:568-70, 2007 | To describe reported immunisation status and primary health care utilisation in refugee and migrant young people | Australia | Healthcare utilization | 68 (41%) had a named general practitioner and 66 (40%) reported seeing a doctor in Australia |
| Yun K, Fuentes-Afflick E, Curry LA, et al.: Parental Immigration Status is Associated with Children’s Health Care Utilization: Findings from the 2003 New Immigrant Survey of US Legal Permanent Residents. Maternal and child health journal 17:1913-21, 2013 | To examine the association between parental immigration status and child health and health care utilization | America | Utilization of annual preventive care and dental care | Children whose parents had been either undocumented or temporary residents were most likely to have a delayed preventive annual exam (18.2 and 18.7 %, respectively). |
| Beiki O, Karimi N, Mohammadi R: Parental educational level and injury incidence and mortality among foreign-born children: a cohort study with 46 years follow-up. Journal of injury & violence research 6:37-43, 2014 | To compare the risk of fatal and non-fatal unintentional injuries among migrant children with lowest parental education with that among children with highest parental education. | Europe | Hospitalization and death due to unintentional injury | Children whose parents had <9 years education (compared with 13 or more) had higher risk of non-fatal (1.08, 95% CI 1.06-1.11) and fatal (HR 1.81 , 95% CI 1.46-2.26) accidents |
